# Characterization of Co-infection With Fowl Adenovirus Serotype 4 and 8a

**DOI:** 10.3389/fmicb.2021.771805

**Published:** 2021-11-03

**Authors:** Jingyi Liu, Xinjin Shi, Lu Lv, Kai Wang, Zhiwei Yang, Yunzhang Li, Hongjun Chen

**Affiliations:** Shanghai Veterinary Research Institute, Chinese Academy of Agricultural Sciences, Shanghai, China

**Keywords:** fowl adenovirus, FAdV-4, FAdV-8a, co-infection, pathogenicity

## Abstract

Fowl adenoviruses (FAdVs), which are distributed worldwide, have caused considerable economic losses to poultry farms. Co-infection with FAdVs and other avian pathogens has been reported previously. However, the pathogenicity of different serotypes of FAdVs causing co-infection remains unclear. Herein, strain HN from FAdV species C serotype 4 (FAdV-4) and strain AH720 from species E serotype 8a (FAdV-8a) were used to assess the pathogenicity of their co-infection in specific-pathogen-free (SPF) chickens. Compared with chickens infected with FAdV-4 alone, those co-infected with FAdV-4 and FAdV-8a showed similar clinical symptoms, mortality rates and degree of tissue lesions, and notably decreased viral loads of HN. Conversely, the viral loads of AH720 increased markedly in the co-infection group compared with that in chickens infected with AH720 strain alone. Increased viral loads of AH720 in the liver were suspected to contribute to the pathogenicity of chickens co-infected with the HN and AH720 strains. This was further investigated by histopathology and terminal deoxynucleotidyl transferase dUTP nick-end labeling (TUNEL) staining analyses. Collectively, these data indicated that co-infection with FAdV-4 and FAdV-8a suppresses the replication and proliferation of FAdV-4 but enhances the replication and proliferation of FAdV-8a in chicken liver. This study will provide valuable information for the further investigation of the interactions between FAdV-4 and FAdV-8a during co-infection.

## Introduction

Fowl adenoviruses (FAdVs) are non-enveloped double-stranded DNA viruses belonging to the genus *Aviadenovirus*, the family Adenoviridae (Besson et al., 2020). FAdVs are classified into five species (FAdV-A to -E) and 12 serotypes (FAdV-1 to -8a and -8b to -11) (Hess, 2000). Among these, FAdV-A and FAdV-B include FAdV-1 and FAdV-5, respectively, FAdV-C includes FAdV-4 and FAdV-10, FAdV-D includes FAdV-2, FAdV-3, FAdV-9, and FAdV-11, and FAdV-E includes FAdV-6, FAdV-7, FAdV-8a, and FAdV-8b (Fauquet et al., 2005).

Fowl adenoviruses are transmitted horizontally and vertically (Chandra et al., 2000; Grafl et al., 2012), and 3- to 5-week-old broilers are highly susceptible to infection with FAdVs (Shah et al., 2017). Some FAdVs can cause various clinical symptoms, such as hydropericardium-hepatitis syndrome (HHS), inclusion body hepatitis (IBH), and gizzard erosion (Kajan et al., 2013; Zhao et al., 2018; Harrach et al., 2019). HHS is characterized by pericardial effusion and an enlarged liver with petechial hemorrhages in broilers (Ganesh et al., 2002). FAdV-4 is the major causative agent of HHB, which was first reported in Pakistan in 1987 and soon spread worldwide (Yu et al., 2018). Recently, FAdV-4 has emerged as an important pathogen in chickens. Since 2015, outbreaks of HHS caused by FAdV-4 have occurred in many chicken farms in China; this has resulted in an extremely high mortality in chickens (Zhao et al., 2015, 2018; Zhang et al., 2016; Pan et al., 2017c). IBH is characterized by an enlarged liver with hepatic necrosis and eosinophilic or basophilic intranuclear inclusion bodies in hepatocytes; it can be caused by FAdV-2, 8a, 8b, and 11 (Ojkic et al., 2008; Mittal et al., 2014). The presence of FAdV-8a and novel FAdV-E has been reported in poultry farms in China (Chen et al., 2020; Lv et al., 2021).

Recently, several studies have reported co-infections with FAdVs and other pathogens. Yu et al. (2019) reported that the infection rate of FAdV-4 has reached 65.2% in 36 farms in Shandong province, China, and co-infection with FAdV-4 and avian influenza virus (AIV), infectious bursal disease virus (IBDV), and chicken infectious anemia virus (CIAV) were found to be common in these samples. Co-infections with FAdVs and CIAV in broilers have been reported in India as well (Brown Jordan et al., 2019). More recently, Yan et al. (2020) reported the co-infection with FAdV-4 and avian orthoreovirus (ARV) in broilers, and that the co-infection rate in ARV-positive samples reached 63%. Moreover, PCR-based methods, combined with the restriction enzyme analysis of chicken samples have revealed that co-infections with different serotypes of FAdVs exist frequently in broiler chickens (Meulemans et al., 2001; Rahul et al., 2005; Mittal et al., 2014). However, little is known about the pathogenicity of different serotypes of FAdVs during co-infection.

In our previous studies, the FAdV-4 strain HN and FAdV-8a strain AH720, isolated from chickens from poultry farms in Hunan and Anhui provinces, respectively, were identified and characterized (Wang et al., 2019; Lv et al., 2021). In the present study, we aim to establish a chicken model of co-infection with both these stains to investigate the interactions between FAdV-4 and FAdV-8a in specific-pathogen-free (SPF) chickens. This may provide valuable information for further investigations of the interactions between FAdV-4 and FAdV-8a during co-infection.

## Materials and Methods

### Cells, Viruses, and Animals

The chicken liver hepatocellular carcinoma cell line LMH was purchased from the American Type Culture Collection (ATCC); LMH cells were cultured in DMEM/F12 (Gibco, NY, United States) supplemented with 10% fetal bovine serum (FBS) (Gibco). The FAdV-4 strain HN and FAdV-8a strain AH720 were isolated as described previously (Wang et al., 2019; Lv et al., 2021) and allowed to replicate in LMH cells. SPF chickens were purchased from Merial Vital Laboratory Animal Technologies Co., Ltd. (Beijing, China). All animal experiments were performed with strict adherence to the guidelines for animal use with approval from Shanghai Laboratory Animal Management Committee and the Animal Care and Use Committee of Shanghai Veterinary Research Institute, Chinese Academy of Agricultural Sciences (permit number: SYXK 2020-0027).

### Co-infection With Fowl Adenovirus Serotype 4 and 8a in Specific-Pathogen-Free Chickens

The pathogenicity of FAdV-4 and FAdV-8a during co-infection with these strains was demonstrated in the SPF chickens. First, 52 3-week-old SPF chickens were randomly divided into four groups (*n* = 13 per group). The chickens in group I and group II were challenged intramuscularly with 100 μl of 10^5^ TCID_50_ of strain HN and 100 μl of 10^5^ TCID_50_ of strain AH720, respectively. The chickens in group III were challenged intramuscularly with a mixture of 100 μl of 10^5^ TCID_50_ of strain HN and 100 μl of 10^5^ TCID_50_ of strain AH720. The chickens in group IV were intramuscularly challenged with 100 μl of PBS. At 3 days post-challenge, three chickens from each group were sacrificed. Tissue samples, including tissues from the liver, pancreas, kidney, spleen, lung, duodenum, jejunum, rectum, and cecum, were collected. These samples were divided into two parts. One part was used for DNA extraction to monitor the viral loads and the second part was fixed in 10% neutral formalin. The remaining 10 chickens from each group were monitored daily and scored for clinical signs for 14 days, as described previously (Zhao et al., 2015). The scoring scheme was as follows: 0 for normal, 1 for mild depression, 2 for severely depressed, 3 for paralysis/prostration, and 4 for death. The survival of the remaining chickens was monitored.

### Quantification of Viral Loads in Tissues

The viral loads in the tissues of infected chickens were determined using TaqMan probe fluorescence quantitative polymerase chain reaction (qPCR). The total DNA was isolated from the liver, pancreatic, kidney, spleen, lung, duodenal, jejunal, rectal, and cecal tissue samples. The FAdV-4 *hexon* gene (1293–1417 nt) was used as an indicator for the presence of HN strain DNA and the FAdV-8a *fiber* gene (836–904 nt) was used as an indicator for the presence of AH720 strain DNA, as described in previous studies (Wang et al., 2019; Lv et al., 2021). qRT-PCR was performed on an Applied Biosystem 7500 Fast instrument with the following cycling conditions: 95°C (5 min), 40 cycles at 95°C (10 s) and 60°C (15 s) and 60°C (30 s). The standard curves were generated, based on which the quantity of the viral DNAs in the tissue samples were calculated.

### Histopathology Examination

The liver, kidney, lung, and spleen tissue samples collected from three chickens in each group were fixed in 10% neutral-buffered formalin for histopathological examination. The samples were routinely dehydrated, embedded in paraffin wax, and then sectioned for hematoxylin and eosin (H&E) staining. The tissue samples were examined under a Nikon microscope equipped with an Olympus DP25 camera. The histopathological lesions were assessed using the following scoring scheme: 0 for no lesions, 1–3 for mild lesions, 4–6 for moderate lesions, and 7–10 for severe lesions (Zhao et al., 2015).

### Immunofluorescence Assay

An immunofluorescence assay (IFA) was performed to investigate the distribution of the viral particles in the livers of chicken infected with the mixture of the HN and AH720 strains. The slides of livers from chickens infected with the HN and AH720 strains were serially cut, blocked using 2% BSA for 1 h, and incubated overnight at 4°C with FAdV-4 Hexon 1B4 monoclonal antibody (1:1000 dilution) and FAdV-8a Fiber polysera (1:1000 dilution) (both prepared by our lab), respectively. Following three washes in PBS buffer, the slides were incubated with Alexa Fluor 488-conjugated goat anti-mouse antibody and Alexa Fluor 594-conjugated goat anti-mouse IgG, respectively. Whole-slide images were captured using the Pannoramic confocal 3D HISTECH system and analyzed by PanoramaStudio Pro software.

### Terminal Deoxynucleotidyl Transferase dUTP Nick-End Labeling Staining

Terminal deoxynucleotidyl transferase (TdT)-mediated dUTP nick-end labeling (TUNEL) assay was performed to evaluate the degree of apoptosis in liver samples from the chickens in the different infection groups, according to the manufacturer’s instructions. The samples were incubated with 50 μl of TUNEL reaction mixture (TdT and fluorescein – dUTP) at 37°C for 60 min in a humid atmosphere. The TUNEL staining intensity was examined, and images were captured using a Pannoramic confocal 3D HISTECH system and analyzed using the PanoramaStudio Pro software. The TUNEL-positive cells were counted, and the average numbers of these cells were compared.

### Statistical Analysis

The data were presented as the means ± SEM. All the data were analyzed using the Prism 7 software (GraphPad, La Jolla, CA, United States). A paired two-tailed Student’s *t*-test was performed to compare the means of data from two groups. The differences were considered statistically significant at *p*-values < 0.01 or < 0.05.

## Results

### Pathogenicity of Chickens Co-infected With the HN and AH720 Strains

**FIGURE 1:**
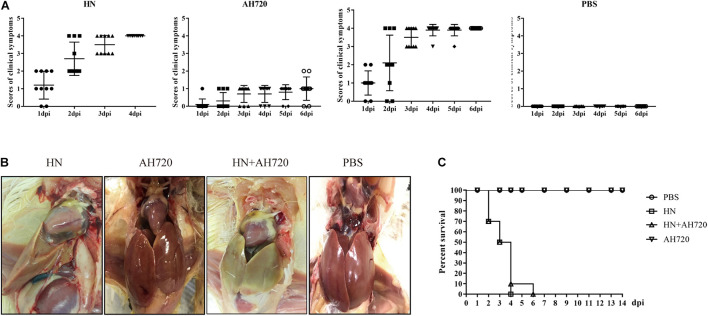
Pathogenicity of chickens from different infection groups. **(A)** Clinical scores of chickens in four different infection groups. Chickens were randomly divided into four groups and challenged with HN strain (group I), AH720 strain (group II) and a mixture of HN and AH720 strains (group III), and PBS (group IV). Clinical scoring: 0 for normal, 1 for mild depression, 2 for severely depressed, 3 for paralysis/prostration, and 4 for death. **(B)** Gross lesions of chickens in four different infection groups. The livers of chickens from groups I and III both showed enlarged yellow and pericardial effusion. A chicken in group II showed a liver with hemorrhages and a chicken from group IV showed a normal liver. **(C)** Survival rate of the chickens in four different groups. No death was found in the chickens infected with AH720 strain. The mortality rate of chickens challenged with HN strain reached 100% within 4 days post-challenge. However, 100% mortality rate in chickens from group III was within 6 days when co-infected with HN and AH720 strains.

To determine the pathogenicity of chickens co-infected with HN and AH720 strains, the chickens were first randomly divided into four groups and then challenged with the HN strain (group I), AH720 strain (group II), mixture of the HN and AH720 strains (group III), and PBS (group IV). During the infection period, the clinical scores varied for the chickens from the different infection groups. The clinical scores for AH720 infection were less than those for HN infection and those for HN + AH720 infection (Figure 1A). The chickens from groups I and III showed typical symptoms of HHS and IBH, with enlarged yellow and hemorrhagic liver and pericardial effusion (Figure 1B). The chickens from group II showed mild symptoms with minor liver hemorrhage (Figure 1B). The control chickens (those from group IV) showed no clinical symptoms.

Further, the mortality rates of the chickens from the different groups were investigated. The mortality rates of the chicken from groups I and III were both 100%, while no death was observed in the cases of the chickens from groups II and IV (Figure 1C). Notably, the mortality rate of the chicken from group I reached 100% within 4 days post-challenge. However, the mortality rate of the chickens from group III reached 100% at 6 days post-challenge (Figure 1C). These data suggested that co-infection with the HN and AH720 strains was slightly less lethal to the chickens than the infection with the HN strain alone.

### Quantification of the Viral DNA Using TaqMan Probe RT-PCR

To investigate the distribution and viral loads of the HN and AH720 strains in infected chickens, viral copy numbers in different tissues from the FAdV-infected chickens were determined at 3 days post-challenge using the previously established TaqMan probe RT-PCR for FAdV-4 and FAdV-8a (Wang et al., 2019; Lv et al., 2021), respectively.

**FIGURE 2:**
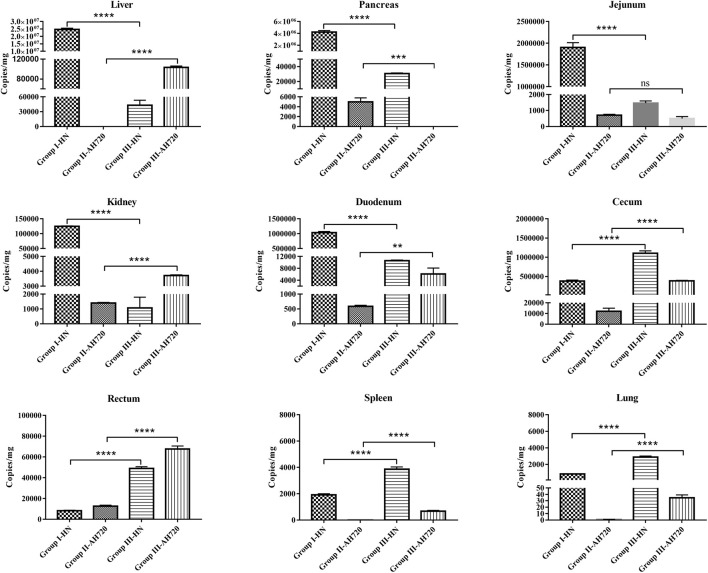
Viral loads in different infected tissues. Tissue samples from liver, pancreas, jejunum, kidney, duodenum, cecum, rectum, spleen, and lung were collected from chickens at 3 days post-challenge. Viral DNA was detected using TaqMan quantitative real-time PCR. The error bars indicate SEM. ns represents not significant, **P < 0.01, ***P < 0.001, ****P < 0.0001 (Student’s *t*-test).

In chickens from group I, the HN strain was detected in all the tissue samples, with the highest counts in the liver (approximately 2.5 × 10^7^ copies/mg), followed by the pancreas, jejunum, kidney, duodenum, and cecum (Figure 2). In the chickens from group II, the AH720 strain was detected in most tissue samples, but the viral loads were relatively low, with the highest viral load being observed in the cecum (approximately 1 × 10^4^ copies/mg) (Figure 2). However, the viral loads of the HN and AH720 strains in the chickens from group III differed markedly from these in the chickens from group I and group II. On one hand, the viral loads of the HN strain in the liver, pancreas, jejunum, kidney, and duodenum in the chickens from group III were significantly lower than those in the corresponding organs of chickens from group I. On the other hand, the viral loads of the HN strain in the cecum, rectum, spleen, and lungs from the chickens in group III were higher than those in the corresponding organs from the chickens in group I (Figure 2). In addition to the pancreas and jejunum, a significant increase in the viral load of the AH720 strain was observed in all the tested tissue samples from the chickens in group III, compared with that in the tested tissue sections from the chickens in group II (Figure 2). These results suggested that the interaction between the HN and AH720 strains may influence the viral replication and proliferation of the HN and AH720 strains in different tissues.

### Histopathology

**FIGURE 3:**
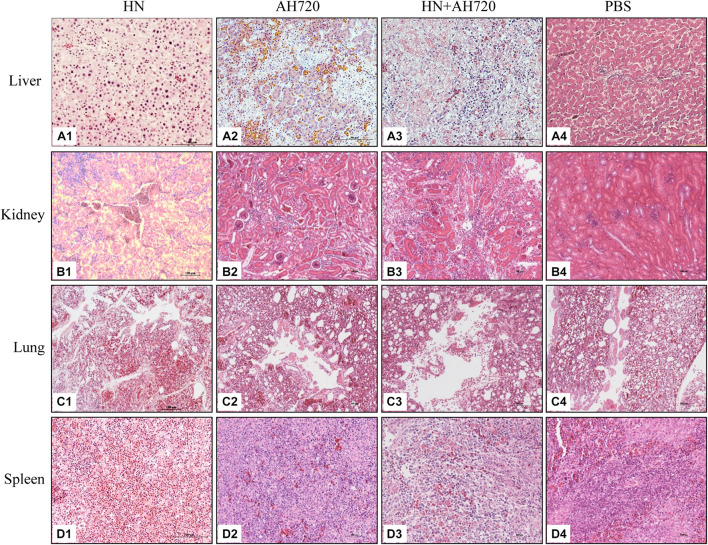
Histological examination of tissue samples from the chickens in different infection groups. **(A1,B1,C1,D1)** H&E staining of liver, kidney, lung, and spleen samples from the chickens infected with HN strain alone. **(A2,B2,C2,D2)** H&E staining of liver, kidney, lung, and spleen samples from the chickens infected with the AH720 strain alone. **(A3,B3,C3,D3)** H&E staining of liver, kidney, lung, and spleen samples from the chickens co-infected with HN and AH720 strains. **(A4,B4,C4,D4)** The normal tissue sample staining in the chickens of the PBS control.

At 3 days post-challenge, the liver, kidney, lung, and spleen tissues from the chickens in the four different groups were fixed for histopathological analysis. Pathological lesions were observed in various tissues of chickens in the different infection groups (Figure 3). The liver tissues from the chickens in group I showed severe liver lesions and presented typical basophilic inclusions with many infiltrating lymphocytes (Figure 3A1). Hepatocyte necrosis was found in the liver tissues of the chickens from group II (Figure 3A2). Lymphocyte infiltration and hepatocyte necrosis were observed in the liver tissues of the chickens from group III (Figure 3A3). No histological changes were observed in the livers of the chickens from the control group (Figure 3A4).

Renal tubular structural disorder was observed in the kidney tissues from chickens in all the three infection groups (Figures 3B1–B3). Inflammatory exudation was observed in the kidneys of the chickens from groups I and III (Figures 3B1,B3). Severe renal hemorrhage was seen in the kidneys of the chickens from group I (Figure 3B1). No histological changes were observed in the kidneys of the chickens from the control group (Figure 3B4).

Structural disorder of the pulmonary bronchus, inflammatory exudation, and alveolar rupture were observed in the lung tissues from the chickens in group I (Figure 3C1). The lung tissues from the chickens in group II were relatively normal (Figure 3C2). Desquamation of pulmonary epithelial cells was observed in the lung tissues from the chickens in group III (Figure 3C3). No significant histological changes were observed in the lung tissues from the chickens in the control group (Figure 3C4).

The number of lymphocytes was reduced in the spleen tissues from the chickens in group I (Figure 3D1). Splenic hemorrhage was observed in the spleen tissues from the chickens in group II (Figure 3D2). Splenic hemorrhage and reduced lymphocytes were observed in the spleen tissues from the chickens in group III (Figure 3D3). No significant histological changes were observed in the spleen tissues from the chickens in the control group (Figure 3D4).

**FIGURE 4:**
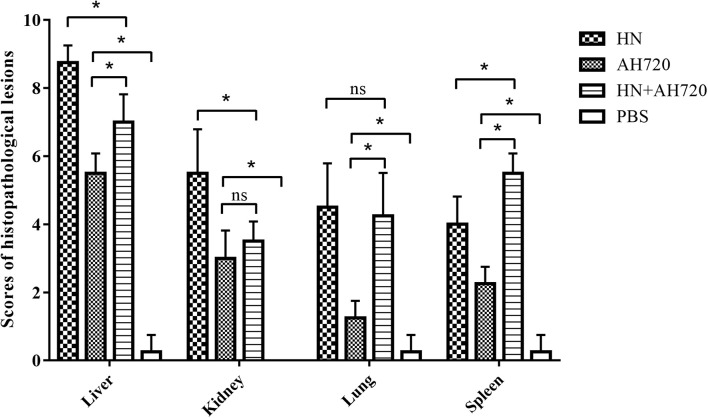
Scores of histopathological lesions in sampled tissues of chickens in different infection groups. Lesion scoring: 0 for no lesions, 1-3 for mild lesions, 4-6 for moderate lesions, and 7-10 for severe lesions. The error bars indicate SEM. ns represents not significant, *P < 0.05 (Student’s *t*-test).

Compared with the control groups, the histopathological lesions in the liver, kidney, lung, and spleen tissues from the chickens in the infection groups were notable (Figure 4). Among the four sampled tissues, the liver tissue showed the highest histopathological scores; the highest histopathological scores were observed for the livers from the chickens in group I (Figure 4).

### Immunofluorescence Assay

**FIGURE 5:**
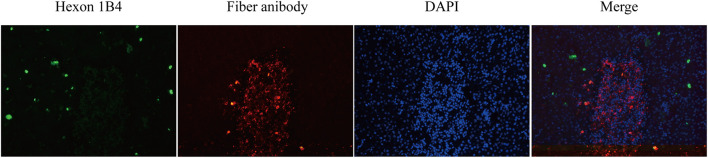
Distribution of viruses in liver of the chickens co-infected with HN and AH720 strains. The slides of liver samples were blocked in 2% BSA and incubated with previously prepared FAdV-4 Hexon 1B4 monoclonal antibody and FAdV-8a Fiber polysera, followed by incubation with Alex Fluor 488 and Alex Fluor 594-conjugated secondary antibodies, respectively. Images were captured by Pannoramic confocal 3D HISTECH system and analyzed by PanoramaStudio Pro software.

The liver tissues collected from the chickens in group III were serially sectioned to investigate the distribution of the viruses in the livers of chickens co-infected with the HN and AH720 strains by IFA. The presence of the HN strain was seen as a green color that was detected by incubation with FAdV-4 Hexon antibody, and subsequently, incubation with Alexa Fluor 488-conjugated secondary antibody. The presence of the AH720 strain was seen as a red color that was detected by incubation with FAdV-8a Fiber antibody, and subsequently, incubation with Alexa Fluor 594-conjugated secondary antibody. IFA analysis of the liver slides revealed that in the chickens co-infected with the HN and AH720 strains, the HN strain presented a scattered distribution and the AH720 virus particles were accumulated in the hepatic cells (Figure 5). This result corresponded with that observed from the histopathological examination of the liver tissues from the chickens co-infected with the HN and AH720 strains.

### Apoptosis in the Liver Tissues

**FIGURE 6:**
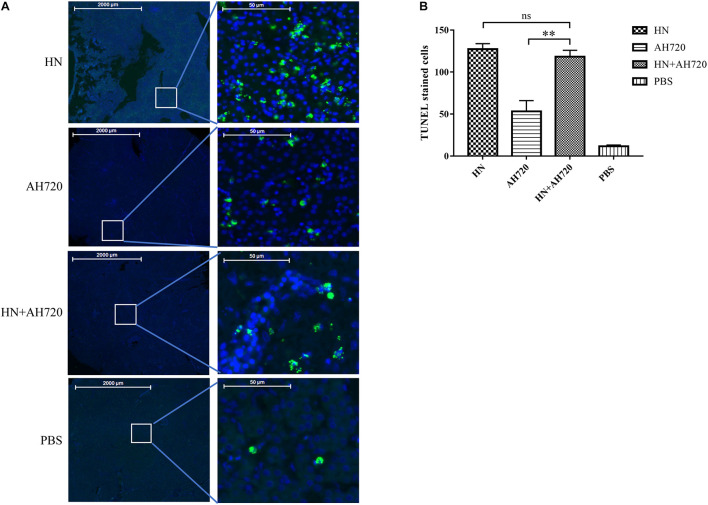
TUNEL staining of livers of the chickens from different infection groups. **(A)** TUNEL staining of livers of chickens from four infection groups. **(B)** Quantification of the TUNEL stained cells. The slides of liver samples were incubated with 50 μl of TUNEL reaction mixture at 37°C for 60 min in a humid atmosphere. The TUNEL intensity was examined and captured by Pannoramic confocal 3D HISTECH system and analyzed by PanoramaStudio Pro software. The TUNEL-positive cells were counted and the average number was compared. The error bars indicate SEM. ns represents not significant, ^∗∗^*P* < 0.05 (Student’s *t*-test).

To further investigate the apoptosis of the liver cells in the chickens from the different infection groups, the collected liver tissue samples were analyzed by TUNEL staining. As shown in Figure 6, the number of TUNEL-positive cells in the livers of chickens from group I was the highest, but this number was not statistically significant compared with that in the livers of chickens from group III. However, the number of TUNEL-positive cells in the livers of chickens from group II was significantly less than that in the livers of chickens from groups I and III. These data indicated that the decrease of the viral load of the HN strain was compensated by the increase of the viral load of the AH720 strain in the liver samples from the chickens in group III, resulting in a similar apoptotic level being observed in the samples from the chickens in group I.

## Discussion

Outbreaks of HHS caused by FAdV infection have been reported in broiler farms in China since 2005 (Pan et al., 2017a; Niu et al., 2018). This highly contagious disease has caused the death of large numbers of chicken, resulting in great economic losses to the poultry industry (Pan et al., 2017b). The pathogenicity of FAdV in cases of co-infection with other pathogens has been previously reported. However, little is known about the pathogenicity of two serotypes of FAdVs during co-infection. Although FAdV-4 was reported as the dominant serotype in chickens infected with FAdVs, isolation of other serotypes of FAdVs, such as FAdV-8a and FAdV-8b, was reported in chicken farms in China (Chen et al., 2019; Cui et al., 2020). In this study, a co-infection model was developed using the FAdV-4 strain HN and FAdV-8a strain AH720 to investigate the co-infection with FAdV-4 and FAdV-8a in chickens. The clinical symptoms, mortality rates, viral loads, and histopathological features of tissues after co-infection were then investigated.

The pathogenicity of different serotypes and strains of FAdVs is different (Grgic et al., 2011; Steer et al., 2015; Wang et al., 2019). We have previously reported that the FAdV-4 strain HN is a virulent strain that could cause 100% mortality in experimentally infected chickens, whereas AH720 is an attenuated strain that caused IBH in, but was not lethal to, chickens (Wang et al., 2019; Lv et al., 2021). To characterize the pathogenicity of the HN and AH720 strains during co-infection with these strains, the chickens were challenged with HN strain (group I), AH720 strain (group II) and a mixture of HN and AH720 strains (group III), and PBS (group IV). Although the mortality rates of the chickens from groups I and III were both 100% (Figure 1C), the trends shown by the change in mortality rates in these two groups were slightly different. The mortality rate in group I reached 100% at 4 days post-challenge, while that in group III was 90% at 4 days post-challenge and reached 100% 2 days later (Figure 1C). In contrast, the mortality rates of chickens in groups III and IV were 0 (Figure 1C), suggesting that the AH720 strain is mildly pathogenic in chickens. This result is consistent with findings from previous studies regarding the pathogenicity of FAdV serotype 8 strains (Grgic et al., 2011; Ruan et al., 2017; Lv et al., 2021). The differences between the mortality rates of the chickens from the four infection groups indicated that interactions between the HN and AH720 strains may slightly influence the outcomes of the disease.

Interactions between FAdVs and other avian pathogens have been previously investigated, and researchers have found that co-infection with FAdVs and other avian pathogens could produce synergistic effects. For instance, co-infection with IBDV and FAdV-4 in chickens could induce immunosuppression and enhance the pathogenicity of FAdV-4 (Xu et al., 2021). Co-infection with *Avibacterium paragallinarum* and FAdV-4 in layer chickens caused more severe clinical symptoms (with 50% mortality), than that caused by infection with FAdV-4 alone (with 40% mortality) (Mei et al., 2020). In addition, co-infection with CIAV and FAdV-4 in chickens can increase the mortality rate and cause severe symptoms, with pericardial effusion and the formation of intranuclear inclusion bodies in hepatocytes (Toro et al., 2000).

In the present study, although the clinical symptoms and mortality rates of the chickens in groups I and III were similar, the viral loads in the tissues of chickens from the different infection groups differed markedly (Figure 2). Compared with the chickens from group I, the viral loads of the HN strain in the liver, pancreas, jejunum, kidney, and duodenum of the chickens from group III were significantly lower, but those in the cecum, rectum, spleen, and lung were relatively higher. Compared with the chickens from group II, all the tested tissues from chickens in group III, except the pancreatic and jejunal tissues, showed significantly higher viral loads of the AH720 strain. Of these changes, it is notable that the biggest decrease of the viral load of the HN strain and increase of the viral load of the AH720 strain were presented in the livers of chickens from group III, compared with the case for the chickens from group I or group II. It is likely that the significant decrease of the viral load of the HN strain in chickens from group III was compensated by the marked increase of the viral loads of the AH720 strain, resulting in similar symptoms and mortality rates being observed in the chickens infected with HN alone.

Further, the histopathological features of tissues of the liver, kidney, lung, and spleen were investigated. Histopathology lesions were observed in all the sampled tissues of the chickens from different infection groups compared with that of the chickens from the control group (Figure 3). Among these, liver tissues of chickens showed the most severe lesions. Notably, hepatocyte accumulation in the liver was observed in the chickens from group III (Figure 3). This is consistent with the results of the IFA analysis, which showed that the HN strain presented a scattered localization, whereas the AH720 strain presented an accumulated localization in the livers of the chickens from group III (Figure 5). Moreover, the TUNEL staining analysis showed that the degree of apoptosis in the livers of the chickens from groups I and III were similar. These data indicated that the decrease in the viral loads of the HN strain in the livers of the chickens infected with the HN and AH720 strains did not affect the symptoms significantly due to the marked increase of the viral loads of the AH720 strain.

## Conclusion

In conclusion, in this study, we established an avian model of co-infection with a FAdV-4 strain and FAdV-8a strain. Co-infection with the HN and AH720 strains decreased the replication and proliferation of the HN strain, and conversely, increased the replication and proliferation of the AH720 strain in chicken livers. The interaction between the HN and AH720 strains allows chickens co-infected with these strains to present similar symptoms and mortality rates as those presented by chickens infected with the HN strain alone. However, the mechanisms underlying the interactions between the HN and AH720 strains during co-infection in chickens require further investigation. To the best of our knowledge, this is the first study that investigates the co-infection with FAdV-4 and FAdV-8a strains experimentally. These findings will lay the foundation for further investigations of the mechanisms underlying co-infection with strains of FAdV-4 and FAdV-8a in chickens.

## Data Availability Statement

The original contributions presented in the study are included in the article/supplementary material, further inquiries can be directed to the corresponding author.

## Ethics Statement

The animal study was reviewed and approved by Shanghai Veterinary Research Institute, Chinese Academy of Agricultural Sciences.

## Author Contributions

HC designed the project. JL, XS, LL, KW, ZY, and YL performed the experiments. HC, JL, and XS analyzed the data. HC and JL wrote the manuscript. All authors read and approved the final manuscript.

## Conflict of Interest

The authors declare that the research was conducted in the absence of any commercial or financial relationships that could be construed as a potential conflict of interest.

## Publisher’s Note

All claims expressed in this article are solely those of the authors and do not necessarily represent those of their affiliated organizations, or those of the publisher, the editors and the reviewers. Any product that may be evaluated in this article, or claim that may be made by its manufacturer, is not guaranteed or endorsed by the publisher.

## References

[B1] Besson S. Vragniau C. Vassal-Stermann E. Dagher M. C. Fender P. (2020). The adenovirus dodecahedron: beyond the platonic story. *Viruses* 12:718. 10.3390/v12070718 32630840 PMC7412204

[B2] Brown Jordan A. Blake L. Bisnath J. Ramgattie C. Carrington C. V. Oura C. A. L. (2019). Identification of four serotypes of fowl adenovirus in clinically affected commercial poultry co-infected with chicken infectious anaemia virus in Trinidad and Tobago. *Transbound. Emerg. Dis.* 66 1341–1348. 10.1111/tbed.13162 30817083

[B3] Chandra R. Shukla S. K. Kumar M. (2000). The hydropericardium syndrome and inclusion body hepatitis in domestic fowl. *Trop. Anim. Health Prod.* 32 99–111. 10.1023/A:1005230703093 10726299

[B4] Chen L. Yin L. Peng P. Zhou Q. Du Y. Zhang Y. (2020). Isolation and characterization of a novel fowl adenovirus serotype 8a strain from China. *Virol. Sin.* 35 517–527. 10.1007/s12250-019-00172-7 31792739 PMC7736427

[B5] Chen L. Yin L. Zhou Q. Peng P. Du Y. Liu L. (2019). Epidemiological investigation of fowl adenovirus infections in poultry in China during 2015-2018. *BMC Vet. Res.* 15:271. 10.1186/s12917-019-1969-7 31370846 PMC6676587

[B6] Cui J. Xu Y. Zhou Z. Xu Q. Wang J. Xiao Y. (2020). Pathogenicity and molecular typing of fowl adenovirus-associated with hepatitis/hydropericardium syndrome in Central China (2015-2018). *Front. Vet. Sci.* 7:190. 10.3389/fvets.2020.00190 32411734 PMC7198797

[B7] Fauquet Claude M. Mayo M. A. Maniloff J. Desselberger U. Ball L. A. (2005). *Virus Taxonomy-8th Report of the International Committee on the Taxonomy of Viruses.* Burlington: Elsevier. 10.1016/B978-0-12-249951-7.50004-3

[B8] Ganesh K. Raghavan R. Gowda R. N. Satyanarayana M. L. Suryanarayana V. V. (2002). Purification and characterization of the aetiological agent of hydropericardium hepatitis syndrome from infected liver tissues of broiler chickens. *Trop. Anim. Health Prod.* 34 7–17. 10.1023/A:1013777509538 11887423

[B9] Grafl B. Aigner F. Liebhart D. Marek A. Prokofieva I. Bachmeier J. (2012). Vertical transmission and clinical signs in broiler breeders and broilers experiencing adenoviral gizzard erosion. *Avian Pathol.* 41 599–604. 10.1080/03079457.2012.740614 23237373

[B10] Grgic H. Yang D. H. Nagy E. (2011). Pathogenicity and complete genome sequence of a fowl adenovirus serotype 8 isolate. *Virus Res.* 156 91–97. 10.1016/j.virusres.2011.01.002 21237223

[B11] Harrach B. Tarjan Z. L. Benko M. (2019). Adenoviruses across the animal kingdom: a walk in the zoo. *FEBS Lett.* 593 3660–3673. 10.1002/1873-3468.13687 31747467

[B12] Hess M. (2000). Detection and differentiation of avian adenoviruses: a review. *Avian Pathol.* 29 195–206. 10.1080/03079450050045440 19184805

[B13] Kajan G. L. Kecskemeti S. Harrach B. Benko M. (2013). Molecular typing of fowl adenoviruses, isolated in Hungary recently, reveals high diversity. *Vet. Microbiol.* 167 357–363. 10.1016/j.vetmic.2013.09.025 24139719

[B14] Lv L. Lu H. Wang K. Shao H. Mei N. Ye J. Q. (2021). Emerging of a novel natural recombinant fowl adenovirus in China. *Transbound. Emerg. Dis.* 68 283–288. 10.1111/tbed.13730 32657542

[B15] Mei C. Xian H. Blackall P. J. Hu W. Zhang X. Wang H. (2020). Concurrent infection of Avibacterium paragallinarum and fowl adenovirus in layer chickens. *Poult. Sci.* 99 6525–6532. 10.1016/j.psj.2020.09.033 33248567 PMC7704954

[B16] Meulemans G. Boschmans M. Berg T. P. Decaesstecker M. (2001). Polymerase chain reaction combined with restriction enzyme analysis for detection and differentiation of fowl adenoviruses. *Avian Pathol.* 30 655–660. 10.1080/03079450120092143 19184959

[B17] Mittal D. Jindal N. Tiwari A. K. Khokhar R. S. (2014). Characterization of fowl adenoviruses associated with hydropericardium syndrome and inclusion body hepatitis in broiler chickens. *Virusdisease* 25 114–119. 10.1007/s13337-013-0183-7 24426318 PMC3889237

[B18] Niu Y. Sun Q. Zhu M. Zhao J. Zhang G. Liu X. (2018). Molecular epidemiology and phylogenetic analysis of fowl adenoviruses caused hydropericardium outbreak in China during 2015. *Poult. Sci.* 97 803–811. 10.3382/ps/pex338 29370402

[B19] Ojkic D. Martin E. Swinton J. Vaillancourt J. P. Boulianne M. Gomis S. (2008). Genotyping of Canadian isolates of fowl adenoviruses. *Avian Pathol.* 37 95–100. 10.1080/03079450701805324 18202956

[B20] Pan Q. Yang Y. Shi Z. Liu L. Gao Y. Qi X. (2017c). Different Dynamic Distribution in chickens and ducks of the hypervirulent, novel genotype fowl adenovirus serotype 4 recently emerged in China. *Front. Microbiol.* 8:1005.10.3389/fmicb.2017.01005 10.3389/fmicb.2017.01005 28634474 PMC5459905

[B21] Pan Q. Liu L. Gao Y. Liu C. Qi X. Zhang Y. (2017a). Characterization of a hypervirulent fowl adenovirus 4 with the novel genotype newly prevalent in China and establishment of reproduction infection model of hydropericardium syndrome in chickens. *Poult. Sci.* 96 1581–1588. 10.3382/ps/pew431 28339951

[B22] Pan Q. Yang Y. Gao Y. Qi X. Liu C. Zhang Y. (2017b). An inactivated novel genotype fowl adenovirus 4 protects chickens against the hydropericardium syndrome that recently emerged in China. *Viruses* 9:216. 10.3390/v9080216 28786949 PMC5580473

[B23] Rahul S. Kataria J. M. Senthilkumar N. Dhama K. Sylvester S. A. Uma R. (2005). Association of fowl adenovirus serotype 12 with hydropericardium syndrome of poultry in India. *Acta Virol.* 49 139–143. 16047743

[B24] Ruan S. F. Zhao J. Ren Y. C. Feng J. L. Zhang G. Z. (2017). Phylogenetic analyses of fowl adenoviruses (FAdV) isolated in China and pathogenicity of a FAdV-8 isolate. *Avian Dis* 61 353–357. 10.1637/11671-050817-RegR 28956998

[B25] Shah M. S. Ashraf A. Khan M. I. Rahman M. Habib M. Chughtai M. I. (2017). Fowl adenovirus: history, emergence, biology and development of a vaccine against hydropericardium syndrome. *Arch Virol.* 162 1833–1843. 10.1007/s00705-017-3313-5 28283816

[B26] Steer P. A. Sandy J. R. O’rourke D. Scott P. C. Browning G. F. Noormohammadi A. H. (2015). Chronological analysis of gross and histological lesions induced by field strains of fowl adenovirus serotypes 1, 8b and 11 in one-day-old chickens. *Avian Pathol.* 44 106–113. 10.1080/03079457.2015.1007919 25609454

[B27] Toro H. Gonzalez C. Cerda L. Hess M. Reyes E. Geissea C. (2000). Chicken anemia virus and fowl adenoviruses: association to induce the inclusion body hepatitis/hydropericardium syndrome. *Avian Dis.* 44 51–58. 10.2307/1592507 10737644

[B28] Wang K. Sun H. Li Y. Yang Z. Ye J. Chen H. (2019). Characterization and pathogenicity of fowl adenovirus serotype 4 isolated from eastern China. *BMC Vet. Res.* 15:373. 10.1186/s12917-019-2092-5 31660972 PMC6816224

[B29] Xu A. H. Sun L. Tu K. H. Teng Q. Y. Xue J. Zhang G. Z. (2021). Experimental co-infection of variant infectious bursal disease virus and fowl adenovirus serotype 4 increases mortality and reduces immune response in chickens. *Vet. Res.* 52:61. 10.1186/s13567-021-00932-y 33926543 PMC8082832

[B30] Yan T. Zhu S. Wang H. Li C. Diao Y. Tang Y. (2020). Synergistic pathogenicity in sequential coinfection with fowl adenovirus type 4 and avian orthoreovirus. *Vet. Microbiol.* 251:108880. 10.1016/j.vetmic.2020.108880 33091795

[B31] Yu G. Lin Y. Dou Y. Tang Y. Diao Y. (2019). Prevalence of fowl adenovirus serotype 4 and co-infection by immunosuppressive viruses in fowl with hydropericardium hepatitis syndrome in shandong province, China. *Viruses* 11:517. 10.3390/v11060517 31195615 PMC6631144

[B32] Yu G. Wang Y. Zhang M. Lin Y. Tang Y. Diao Y. (2018). Pathogenic, phylogenetic, and serological analysis of group i fowl adenovirus serotype 4 SDSX isolated from Shandong, China. *Front. Microbiol.* 9:2772. 10.3389/fmicb.2018.02772 30510548 PMC6252349

[B33] Zhang T. Jin Q. Ding P. Wang Y. Chai Y. Li Y. (2016). Molecular epidemiology of hydropericardium syndrome outbreak-associated serotype 4 fowl adenovirus isolates in Central China. *Virol. J.* 13:188. 10.1186/s12985-016-0644-x 27863494 PMC5116161

[B34] Zhao J. Ruan S. Guo Y. He Z. Xu M. Zhang G. (2018). Serological and phylogenetic analysis indicating prevalence of fowl adenovirus in China. *Vet. Rec.* 182:381. 10.1136/vr.104517 29305398

[B35] Zhao J. Zhong Q. Zhao Y. Hu Y. X. Zhang G. Z. (2015). Pathogenicity and complete genome characterization of fowl adenoviruses isolated from chickens associated with inclusion body hepatitis and hydropericardium syndrome in China. *PLoS One* 10:e0133073. 10.1371/journal.pone.0133073 26167857 PMC4500579

